# The early days of structural biology at the Beijing Institute of Biophysics: In memory of Professor Zhengjiong Lin (1935–2022)

**DOI:** 10.1007/s13238-022-00916-4

**Published:** 2022-04-24

**Authors:** Jia-huai Wang

**Affiliations:** Dana-Farber Cancer Institute, Harvard Medical School, Boston, United States

At the end of 2017, while I was cleaning my apartment in Beijing, a thin journal caught my attention deep in one dusty cabinet. To my surprise, it was an old lab notebook I used 55 years ago! I could not help opening it. Written on the first page was my mentor Zhengjiong Lin’s assignment for my first project: To determine the crystal structure of a di-peptide, Proline-Glycine.

**Figure 1:**
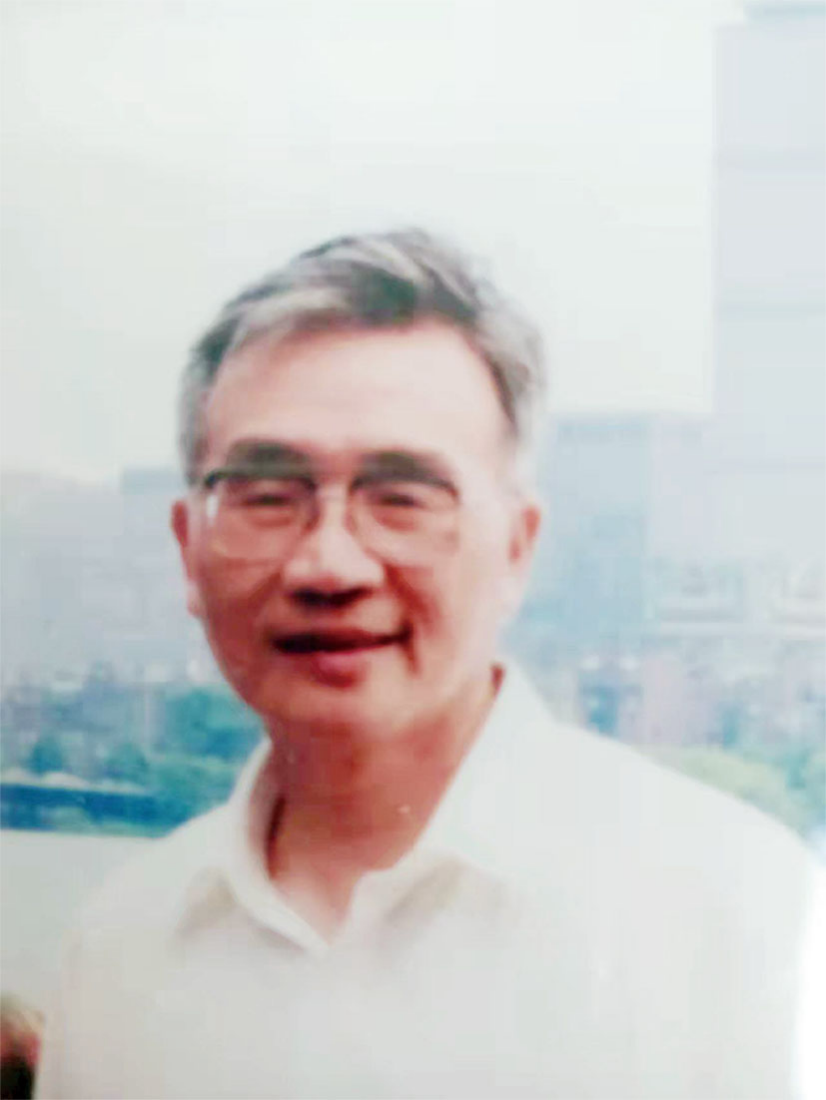
Zhengjiong Lin (1935–2022)

Retired a few years ago from the Chinese Academy of Sciences (CAS), Professor Lin received his initial training in the well-known structural chemistry lab at Peking University (Fig. 1). Immediately after graduating in 1958, he was recruited by the late visionary Professor Shizhang Bei to his newly founded Institute of Biophysics (IBP), one of the earliest research centers in the world for biophysics. Bei’s extraordinary ambition was to one day work out the structure of a protein as one of his new institute’s endeavors. This was the year the whole scientific community witnessed the birth of the first protein structure, myoglobin at 6 Å resolution (Kendrew et al. 1958). The Nobel-winning masterpiece was done at the famous Cavendish laboratory in Cambridge, UK, where modern X-ray crystallography was pioneered by William Bragg. But at the time, literally destitute Zhengjiong had nothing to start with. In scientific terms, it was truly a blank page. But as a down-to-earth person, he knew he had to strive one step at a time. So he went to work at an established X-ray crystallography lab led by Dongcai Liang and Haifu Fan at the nearby Institute of Physics. At that time the Liang/Fan group was one of the best labs in China for carrying out any single crystal structure analysis.

For more than three decades, the IBP did not have its own central building. Instead, the research labs and administration offices were scattered in a dozen different locations. Hence, IBP was affectionately known as “The Ming Tombs,” a reference to the burial sites of ancient emperors outside Beijing. Lin’s lab was on the first floor of the Microbiology Institute building. One had to bow through a side door to reach the street. The cramped room was outfitted with “the best we could find” equipment, essentially just an X-ray generator that was designed for medical usage. It used a two-level pump system to create a vacuum barely good enough for X-ray generation. The X-ray was so weak that to take a diffraction photograph from a big single crystal of a small organic molecule with a dozen atoms would take more than one week. Nowadays, with a modern X-ray synchrotron, acquiring a full data set of 1 million spots from a weakly diffracting crystal of a protein with tens of thousands of atoms can be easily done in just a couple of minutes! Students can sit back and watch the robot-controlled data collection while enjoying some potato chips and a can of Coke. That was certainly not in my wildest dreams when I painfully learned to collect my first data set.

I was the first student Lin recruited, still in my senior year at the University of Science and Technology of China (USTC). I was very reluctant to join the lab, though, because I was fascinated by another field, bioenergetics. Professor Bei was also the Chairman of the Biophysics Department at USTC. He and his vice-chairman, Professor Shuming Shen, insisted that the decision to assign a student to Lin was part of their strategic plan for the institute: To set up a protein crystallography group to initiate structural biology efforts. Bei also invited renowned Professor Chen-lu Tsou from Shanghai Biochemistry Institute to serve as a supervisor for the group. Tsou’s early research at Cambridge University on cytochrome C is considered a first step toward the eventual discovery of a protein structure (Slater 2007). Tsou came to IBP from Shanghai for three months each year. His wife Anna Lin, a physicist, was working at the Physics Institute at CAS. His father-in-law, Siguang Li, was the most famous geologist in China. Tsou, Anna and her father were all academicians at CAS and were the most distinguished science family in China. Tsou’s enlightening chats gave us a good sense of what a protein should look like. And I have been greatly indebted to Tsou for advising me ever since. As a junior faculty member, Lin was pleased to have their support.

What could be useful about working on a bi-peptide? I thought at the time. Even a small protein should consist of about a hundred amino acid residues. Lin advised me to read a few classic papers published by Linus Pauling in the 1950s. The gifted Pauling and his colleagues Robert Corey and Herman Branson proposed that the primary structure of a protein is comprised of α helices and β sheets (Pauling et al. 1951). The conclusion they arrived at was based on a series of experimental crystal structures of small peptide molecules, in particular the observation of a planar peptide bond. We can learn a great deal about the structure of a protein from small peptide structures, Lin explained to me. And it was the reason he designed the experiments. He emphasized the importance of exploring the role that the structurally unusual amino acid proline may play in protein structure, as it was the rationale of our project to determine the crystal structure of Pro-Gly. This method of exploring a big, challenging scientific problem by cleverly designing “small” experiments was enlightening to me.

**Figure 2:**
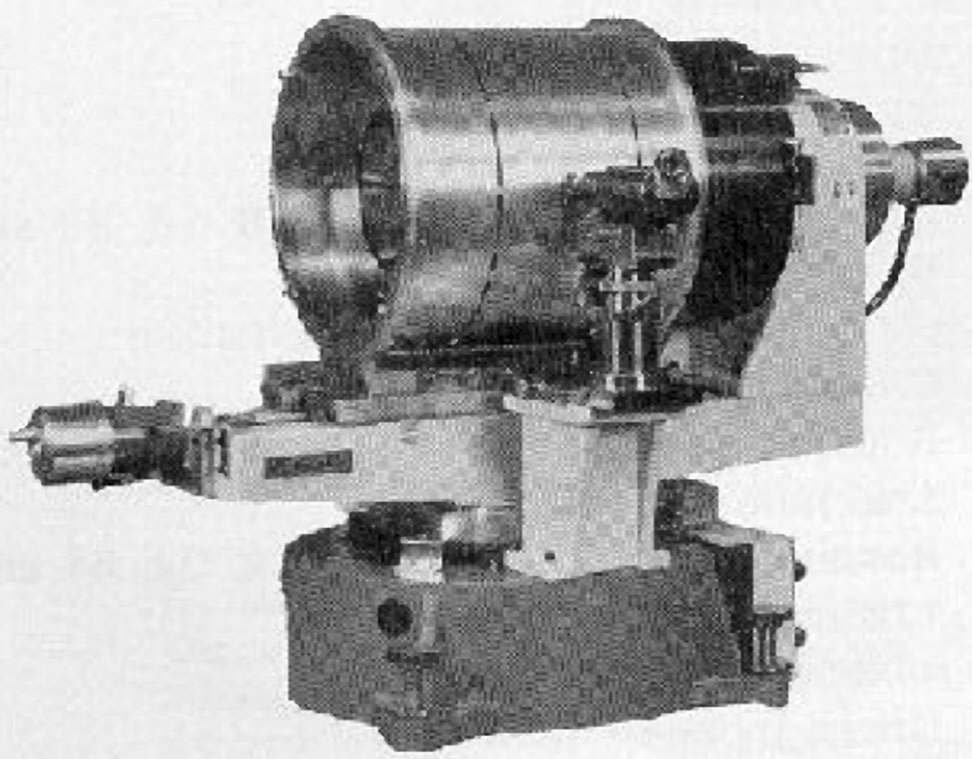
Weissenberg camera

**Figure 3:**
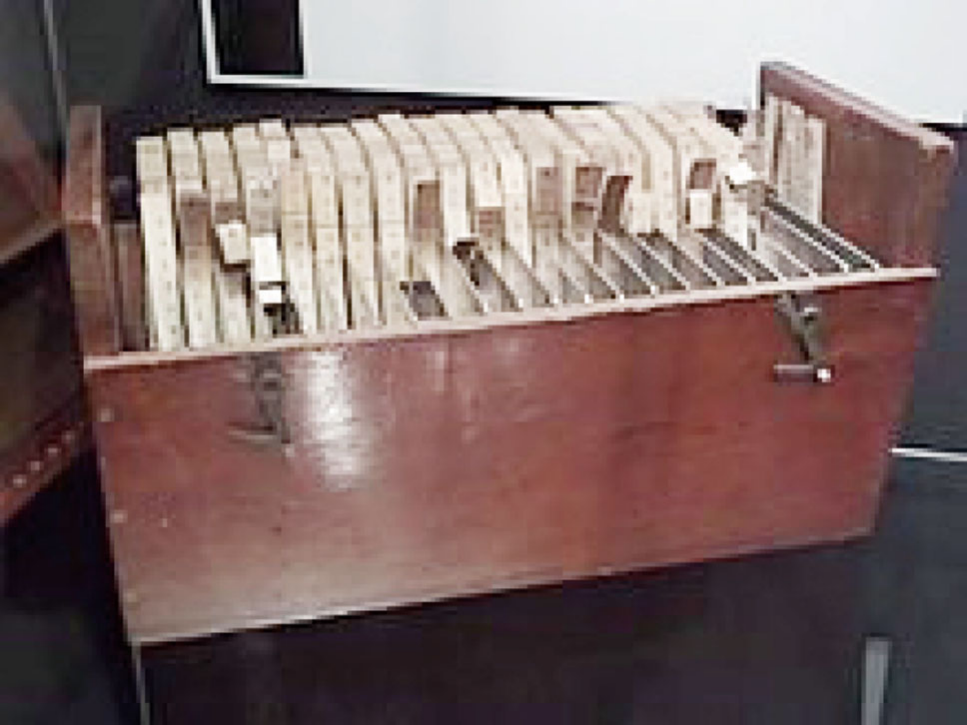
Beevers-Lipson strip

**Figure 4:**
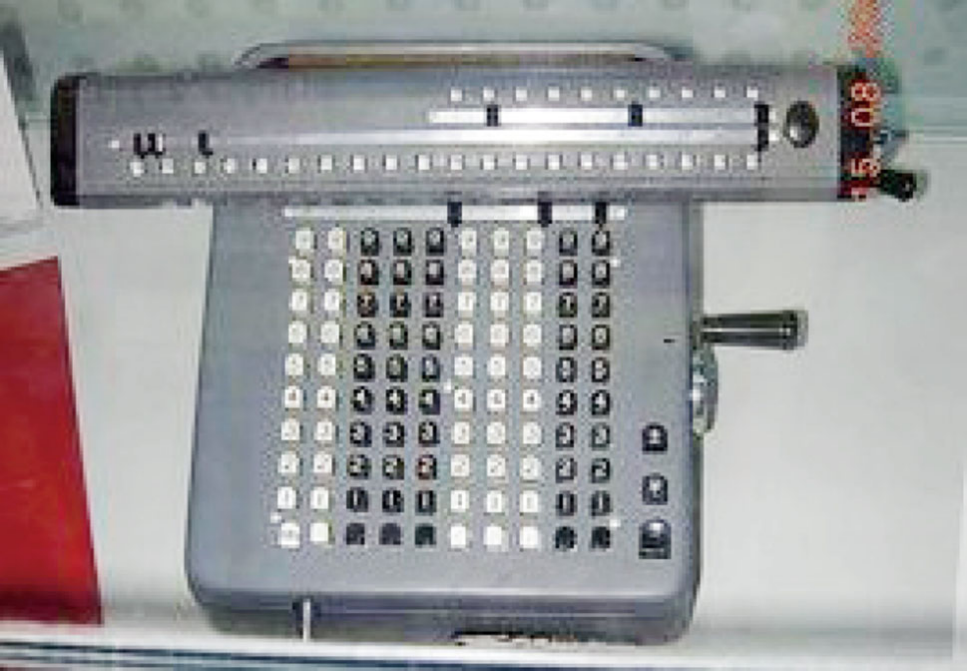
Hand-operated computer

My data collection was actually done in the Liang/Fan lab. Lin taught me hands-on how to use the so-called Weissenberg X-ray diffraction camera (Fig. 2). While a crystal mounted on the camera was rotating around the spindle, a round cassette with film moves back and forth around the cassette such that three-dimensionally distributed diffraction spots can be recorded from X-rays shooting at the crystal. Not only was the recorded diffraction pattern hard to interpret for spots indexing, but I had to measure the intensity for each spot on the film with my naked eye, an extremely boring procedure. One had to first make an intensity scalar: a line of spots with various X-ray exposure time (one second, two seconds, ten seconds, etc.). Then I compared each recorded diffraction spot with these spots of different intensities on the scalar to see what intensity the diffraction spots fell in. This procedure digitizes the whole diffraction data set. In that old notebook I noted that it took me one minute to measure one spot and two weeks to complete the entire data set! The physical principle of X-ray crystallography is the Fourier transform between the three-dimensional distribution of constituent atoms in a crystal and the diffraction pattern of the crystal. In mathematics we calculate the summation of Fourier series. This is a very tedious job. With all the data collected, Lin patiently taught me how to use a so-called Beevers-Lipson strip invented by British crystallographers in the 1930s (Fig. 3) to calculate the Fourier transform, a key step in deriving a crystal structure. Each strip has a series number representing the value points of a sine or cosine function to convert the calculation into Fourier summations. Lin also meticulously taught me how to use crystal symmetry to dramatically reduce the amount of calculation. In 1962, there was no electronic computer to calculate the crystal structure from the data set collected. All we had in the Liang/Fan lab at the Physics Institute was a kind of slide rule and a hand-operated computing machine (Fig. 4). I clearly remember having my first serious migraine attack on the way back from the lab to my dorm while trying to solve the structure of that di-peptide!

**Figure 5:**
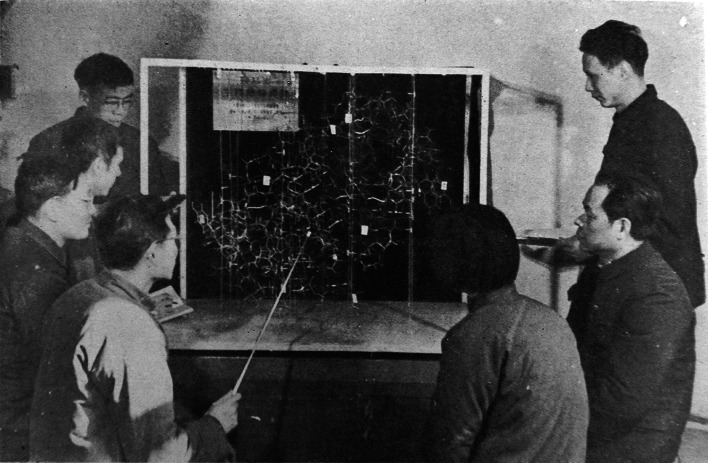
Zhengjiong Lin and colleagues were studying the insulin structure model

With all these seemingly 19th-century technologies, determining the structure of a protein appeared totally unreachable in the foreseeable future. Yet only a couple of years later, in 1966, came extremely exciting news: a group of scientists at the Biochemistry Institute and Organic Chemistry Institute in Shanghai along with those at Peking University in Beijing announced the success of the total chemical synthesis of insulin (龚岳亭 et al. 1965)! Immediately, the most respected structural chemistry professor in China, You-qi-Tang of Peking University realized that it was time to initiate the crystal structure determination of insulin. His suggestion was accepted. It happened just in time, as Dongcai Liang had recently returned from Dorothy Hodgkin’s lab at Oxford University. Liang was the first Chinese scientist to have any practical knowledge of how to solve a protein structure from the Nobel-winning lab. His group at the Physics Institute and Lin’s group at the Biophysics Institute along with those at Peking University were seriously considering a collaboration on the project. All of a sudden, it became clear that we had everything we needed to take on the arduous task: The amino acid sequence of insulin—the first protein sequence—had been published a few years earlier by British Nobel laureate Frederick Sanger (reviewed in (Sanger 1959)). The conditions for crystallizing insulin were known (Hodgkin 1950). An automatic diffraction machine had just been installed at the Physics Institute. And an electronic computer was now available at the Institute of Computer Sciences in CAS. More importantly, a group of young scientists already had enough good knowledge in crystallography to determine the crystal structure of small molecules. We just had to read the literature on what makes protein crystals so unique. I still vividly remember the exciting train trip led by Liang and Lin from Beijing to Shanghai to discuss with the Shanghai Insulin group a potential collaboration on the project. I listened to Liang and Lin on what the major hurdles to solving a protein structure might be, how we could technically conquer it and how we should cooperate. There have been quite a few good memoirs published, in particular an oral history of the insulin structure project, including an interview with Lin (熊卫民 2009). I am not going to repeat here all the unforgettable moments in determining the first protein structure in China—there are too many. Even Dorothy Hodgkin appreciated that it was one of the first dozen protein structures in the world to be discovered (Wang 1998). I should only emphasize that as one of the academic leaders in the group, Lin played a key scientific role in the breakthrough (Fig. 5).

Insulin was one of those rare science projects that was allowed to continue during this chaotic period in China. The project educated a large group of young structural biologists, which became a precious seed for generations to come! As a group leader and later as the chair of Protein Crystallography Department in IBP, Lin further trained a large group of students. Back in the early 1960s, when poor young Zhengjiong Lin had to repair the vacuum system of the medical X-ray machine, his only major equipment, almost every month to get it running properly, he could never have imagined what the booming field of structural biology in China and elsewhere would look like in the future. He was therefore quite pleased to see the achievements of the new generation of structural biologists, many of whom are his former students. A truly modest figure, he was always shy about telling stories about himself, even when IBP personnel visited him for the sake of writing the institute’s history for its 60th anniversary. In 2005, then-IBP director professor Zihe Rao encouraged him to apply for the member of academician at CAS. Like everyone at IBP, Rao truly believed that Lin was more than qualified. However, to Rao’s disappointment, Lin firmly turned him down. Rao did not want to give up and sought my help. He thought that as Lin’s first student and long-time colleague, I might be able to persuade him. I did try very hard in Lin’s home, but it was in vain. The funeral service held for him on January 19, 2022, was a big gathering with Lin’s former students, colleagues and friends from all of the world. Everyone paid their respects. One of my long-time friends, a senior IBP professor, told me that Lin was truly a “good man.” It is certainly a very inornate word for a noble-minded scientist.

Embarrassingly, there was one entry in my lab journal where I noted that due to a careless mistake, I accidently caused Lin to break his eyeglasses. He simply smiled and made no complaint at all. With his passing away, when I look over my old frayed notebook again, his image inevitably appears. His academic rigor, his diligence, his lenience and his undaunted pursuit of science will remain in my mind forever.
